# Feasibility of a noninvasive photoplethysmography-based wearable chest patch for cardiac output assessment during exercise compared with thermodilution right heart catheterization

**DOI:** 10.1038/s41598-026-63092-z

**Published:** 2026-07-22

**Authors:** Bernd Stücker, Alexander Hoss, Jafer Haschemi, Fabian Voß, Amin Polzin, Malte Kelm, Christian Jung, Oliver Maier

**Affiliations:** 1 Division of Cardiology, Pulmonology, and Vascular Medicine, Department of Internal Medicine, Medical Faculty and University Hospital, Heinrich-Heine-University, Moorenstr. 5, 40225 Duesseldorf, Germany; 2 CARID (Cardiovascular Research Institute Düsseldorf), Duesseldorf, Germany

**Keywords:** Wearables, Patch sensor, Hemodynamic monitoring, Heart failure with preserved ejection fraction, Exercise right heart catheterization, Cardiology, Health care, Medical research, Physiology

## Abstract

Hemodynamic monitoring with cardiac output (CO) assessment is usually based on invasive methods that are associated with potential complications. This study compared CO measurements during exercise obtained from a noninvasive photoplethysmography (PPG)-based chest patch monitor with those obtained from invasive thermodilution-based right heart catheterization in patients with heart failure with a preserved ejection fraction (HFpEF). CO was assessed at rest, during passive leg raises, and during 20 Watt (W) of bicycle exercise. Nine patients (67% female) with a mean age of 74.1 ± 12.1 years provided a total of 27 paired measurements. Bland–Altman analysis showed a bias of -0.72 L/min (95% CI − 1.16 to − 0.29) with 95% limits of agreement of − 2.88 to 1.43 L/min and a percentage error of 33%. The CO values obtained by thermodilution and the PPG patch were strongly correlated (r = 0.774; *p* < 0.001) with a concordance of r = 0.88 (*p* = 0.002) for the rest-to-20 W change. In this single-center feasibility study, a noninvasive PPG-based chest patch was feasible during exercise right heart catheterization and tracked exercise-induced changes in cardiac output well. Wearable monitoring may offer a low-risk, complementary approach to hemodynamic assessment, but these findings require confirmation in a larger validation cohort.

**Supplementary Information:**

The online version contains supplementary material available at 10.1038/s41598-026-63092-z.

## Introduction

Hemodynamic monitoring with cardiac output (CO) assessment can provide important information and early recognition of deterioration in patients with heart failure^1,2^. It is important to note that the diagnosis of HFpEF rests fundamentally on the demonstration of elevated left-ventricular filling pressures (e.g. pulmonary capillary wedge pressure), at rest or during exercise, together with signs and symptoms of heart failure, elevated natriuretic peptides and a preserved ejection fraction; cardiac-output assessment in this context serves a complementary role in characterizing the hemodynamic profile and the exercise response rather than being a primary diagnostic criterion. Currently, right heart catheterization is still the main diagnostic tool for defining the different types of heart failure^3^. As these measurements are usually based on invasive methods associated with the possibility of several clinical complications^4^, experts have already recognized the demand for noninvasive tools^3^.

Heart failure with preserved ejection fraction (HFpEF) has gained increasing attention in recent years, showing nearly the same numbers and prognosis as patients with reduced ejection fraction^5^. The causes of HFpEF are diverse. Central adiposity seems to be one of the most important drivers of HFpEF, along with hypertension, atrial fibrillation, and coronary artery disease^6–10^. According to the European Society of Cardiology (ESC), HFpEF is clinically defined as the presence of signs and symptoms of heart failure, raised filling pressures of the left ventricle, elevated levels of natriuretic peptides, and a preserved left ventricular ejection fraction^11^. Exercise right heart catheterization is considered the gold standard for diagnosing HFpEF and precisely determining the hemodynamic situation, especially if echocardiography at rest and laboratory tests are ambiguous^11–14^. However, performing exercise during right heart catheterization (RHC) is invasive and technically challenging, is associated with several complications, and is not universally available^4^.

Wearable devices have gained increasing popularity in recent years. By continuously monitoring physiological parameters, even during exercise, wearables can provide real-time data to healthcare professionals that can be used to guide treatment decisions^15–18^. Although several studies have investigated the feasibility of wearables for monitoring hemodynamics at rest^19–21^, to the best of our knowledge, none have examined their feasibility and validity during exercise.

Therefore, this study aimed to compare CO measurements obtained from a noninvasive photoplethysmography (PPG)-based chest patch wearable monitor with invasive measurements obtained using thermodilution-based exercise RHC in patients with HFpEF, as a feasibility and agreement assessment.

## Methods

### Study design

This was a single-center, prospective, observational, non-randomized feasibility study. From July 2023 to September 2024, patients with a high probability of HFpEF undergoing exercise RHC to confirm their diagnosis were included after informed consent at the Duesseldorf Heart Center. Patients with limited communication, cognitive impairment, or a condition that could interfere with the wearable patch (pacemaker or plaster allergy) were excluded. This study was approved by the institutional ethics committee of the Heinrich-Heine University of Düsseldorf (2022–1981) and conducted in accordance with the Declaration of Helsinki. All participants provided written informed consent.

### Data collection

**Fig. 1:**
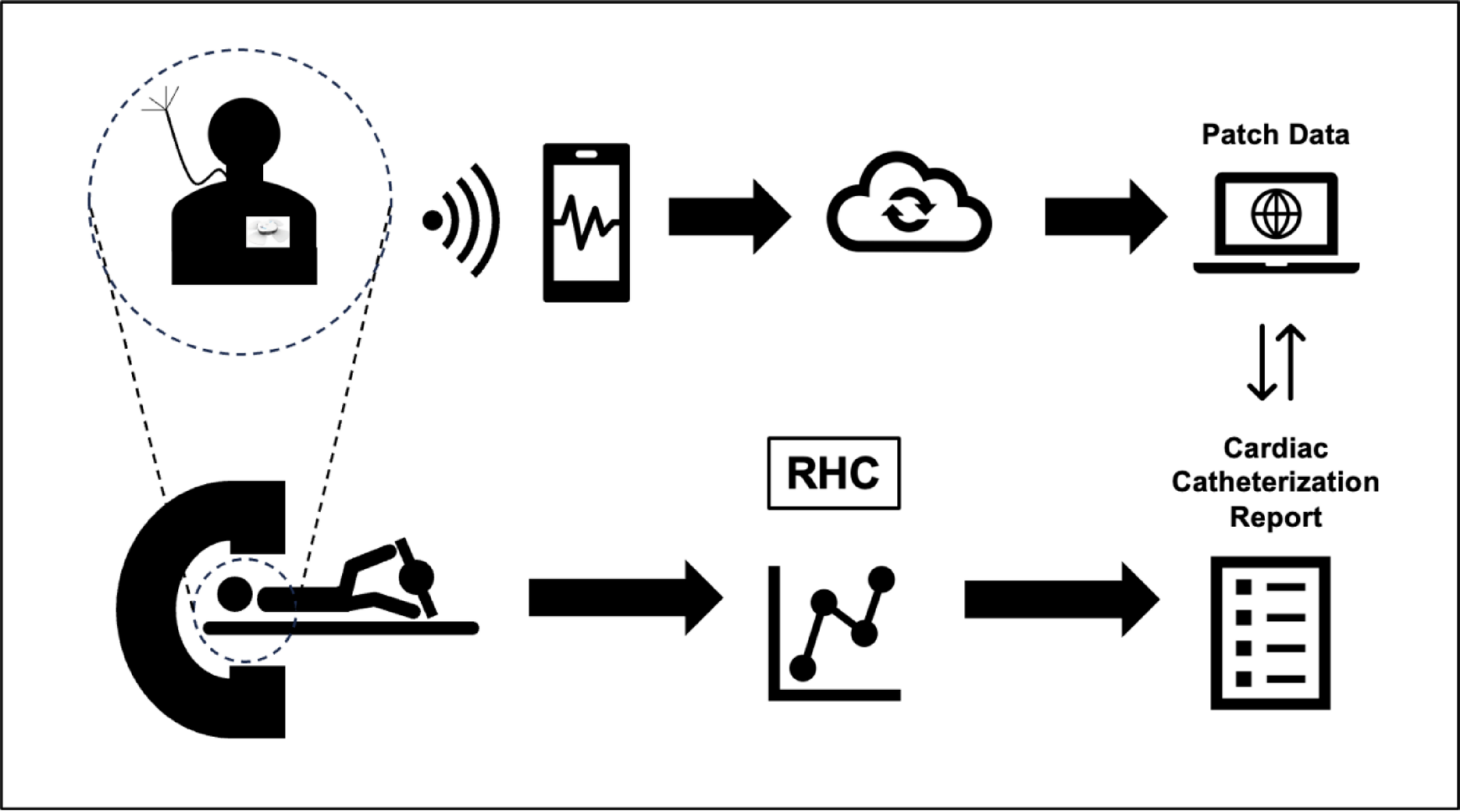
Simultaneous data collection via Biobeat chest patch from the beginning of right heart catheterization (RHC) until the maximum of exercise. Participants performing exercise by bicycle on an ergometer while lying on the table of the cardiac catheterization lab. The access site for the RHC was the right internal jugular vein. 3 stages of exercising: at rest, passive leg raise and 20 Watt of bicycle exercise.

Patients received standard diagnostics by RHC, in addition to vital-sign monitoring using a PPG-based chest monitor patch (Biobeat Technologies Ltd., Petah-Tikva, Israel) (Supplementary Figure S1). Data from the PPG patch were sent via Bluetooth to a mobile phone running the manufacturer’s application, which uploaded the data to the manufacturer’s secure cloud server (Fig. 1). The patch provides automatic real-time monitoring of vital signs every 5 s, including noninvasive blood pressure, heart rate, oxygen saturation, respiratory rate, stroke volume, cardiac output, systemic vascular resistance, skin temperature and single-lead electrocardiography. All cardiac output, stroke volume and systemic vascular resistance values reported for the patch are non-invasive, algorithm-derived estimates rather than direct measurements; for clarity the corresponding tables and figures label these variables as estimated (e.g. CO, estimated L/min).

We gathered data via the chest patch from the beginning of RHC until maximum exercise. Participants performed exercise using a bicycle ergometer while lying on the table of the cardiac catheterization lab. The access site for RHC was the right internal jugular vein (Fig. 1). We conducted three stages: at rest, during passive leg raises, and during 20 W of bicycle exercise, receiving simultaneous hemodynamic data from both the RHC and PPG patches.

### Exercise right heart catheterization

RHC was performed using a regular pulmonary artery (Swan-Ganz) catheter. The physical principle is based on Fick’s principle: the dilution of a cold fluid bolus is measured, and the progression of the temperature curve in the pulmonary artery over time allows calculation of cardiac output. Using a pressure sensor, right atrial pressure, pulmonary arterial pressure and pulmonary capillary wedge pressure (PCWP) were assessed^1^.

At each exercise stage (rest, passive leg raise, 20 W), cardiac output was determined by thermodilution from three sequential manual cold-saline bolus injections (each 10 ml with a temperature of 5 degrees Celsius), and the mean of the three measurements was used as the reference value for that stage; all thermodilution values reported in the text, tables and figures are therefore the mean of three sequential boluses rather than single measurements. As the patch data needed to be uploaded to the data cloud before it was available, the operators of the RHC were blinded to the live patch data.

### Wearable PPG-based chest monitor patch

PPG is a noninvasive optical technique used to detect volumetric changes in blood at the surface of the skin using different wavelengths of light. These physiological volumetric changes in arterial blood signals are associated with cardiac activity. The feasibility of the PPG-based technique was previously demonstrated against arterial lines and RHC^22,23^.

The device estimates cardiac output from the PPG pulse wave form using pulse-wave analysis. In brief, the reflective multi-wavelength PPG signal acquired from the chest is analyzed by an algorithm specifically programmed by the manufacturer Biobeat. Features related to pulse-wave transit time and to the contour of the arterial pulse wave are used to estimate stroke volume on a beat-to-beat basis. Cardiac output is then computed as the product of estimated stroke volume and heart rate, anchored to the patient through an initial blood-pressure calibration. The exact computational algorithm is proprietary to the manufacturer and is therefore not disclosed in full^23^.

To start monitoring, a baseline calibration of the current blood pressure must be performed by cuff measurement prior to data acquisition. The patch values reported for each thermodilution measurement are the mean of the patch records within a defined time window centered on the thermodilution acquisition (the patch samples automatically every 5 s).

### Study objectives and outcome measures

The primary objective of this study was the feasibility assessment and comparison of hemodynamic data obtained from a noninvasive wearable patch sensor during exercise with thermodilution-based hemodynamic monitoring parameters from exercise right heart catheterization. Patch data were described as the mean of records during the three measurements from RHC performed at each exercise level.

### Statistical analysis

Continuous data are described as mean ± standard deviation (SD). Categorical variables are presented as frequencies and percentages. Agreement of the patch data with invasive measurements was assessed using Bland–Altman analysis and Pearson correlation. Because each patient contributed three observations, the Bland–Altman analysis was performed using the method for multiple observations per subject, partitioning the variance of the differences into within- and between-subject components; 95% confidence intervals for the bias and for the limits of agreement (LoA) are reported. We additionally report the percentage error (1.96 × SD of the differences divided by the mean reference cardiac output) following Critchley and Critchley^24–26^ and assessed proportional bias by regressing the differences on the means. Also, we performed stage-specific Bland–Altman analyses for rest, passive leg raise and 20 W, and performed a change (delta) analysis comparing the stage-to-stage changes in cardiac output between the two methods. All tests were 2-tailed, and *p* < 0.05 was considered statistically significant. Analyses were performed using SPSS version 23.0 (IBM SPSS Inc., Chicago, IL, USA).

## Results

### Study cohort

Nine patients were enrolled. The participants had a mean age of 74.1 ± 12.1 years and were predominantly female (n = 6; 67%). All were diagnosed with HFpEF during RHC. The average body mass index (BMI) was 29.6 kg/m^2^. Eight of the nine patients had a BMI ≥ 25 kg/m^2^ (88.9%); of these, four were overweight (BMI 25.0–29.9 kg/m^2^) and four were obese (BMI ≥ 30 kg/m^2^, mainly grade 1), following the WHO classification. Of the total population, 67% had arterial hypertension and 89% had pulmonary hypertension. Four of the nine patients (44%) had coronary artery disease, two had atrial fibrillation, and all had chronic kidney disease (grade 2 or 3) without chronic dialysis (Table 1). The different HFpEF entities are shown in Supplementary Figure S2.

**Table 1:** Baseline characteristics of the study cohort.

Baseline data	
Age, years	74.1 ± 12.1
Gender, female (%)	6 (66.6)
Height, cm	168.3 ± 11.2
Weight, kg	84.4 ± 17.9
Body mass index, kg/m^2^	29.6 ± 3.7
HFpEF (%)	9 (100.0)
Arterial hypertension (%)	6 (66.6)
Pulmonary hypertension (%)	8 (88.9)
Diabetes mellitus (%)	1 (11.1)
Smoking (%)	2 (22.2)
COPD (%)	1 (11.1)
Chronic kidney disease (%)	Grade 2: 4 (44.4); Grade 3: 5 (55.5)
History of stroke or TIA (%)	2 (22.2)
History of myocardial infarction (%)	1 (11.1)
CAD (%)	4 (44.4)
Atrial fibrillation (%)	2 (22.2)
Signs of RCM (%)	2 (22.2)
Medication	
Diuretics (%)	9 (100.0)
SGLT2 inhibitor (%)	8 (88.9)
ß-blocker (%)	4 (44.4)
ACE inhibitor / AT1 blocker (%)	5 (55.6)
Mineralocorticoid-receptor antagonist (%)	0 (0.0)
Sacubitril/Valsartan (%)	0 (0.0)

Values are mean ± SD or n (%). ACE, angiotensin-converting-enzyme; AT1, angiotensin II type-1; CABG, coronary artery bypass graft; CAD, coronary artery disease; COPD, chronic obstructive lung disease; HFpEF, heart failure with preserved ejection fraction; RCM, restrictive cardiomyopathy; SGLT2, sodium/glucose-cotransporter 2; TIA, transient ischemic attack.

Concerning the medication history every patient received diuretics and nearly all received SGLT2-inhibitor therapy (89%), as recommended for HFpEF^27^. Nearly half were medicated with a ß-blocker (44%) and/or an ACE inhibitor / AT1-receptor blocker (56%), while none received a mineralocorticoid-receptor antagonist or sacubitril/valsartan.

From the nine patients, 27 measurements were collected using the PPG chest patch, at three exercise levels: rest (n = 9), passive leg raise (n = 9) and 20 W (n = 9). These were compared with 27 cardiac output measurements generated by RHC, each averaged from three sequential measurements.

### Exercise measurements—descriptive analysis

During the different exercise levels, the patch continuously recorded high-frequency PPG signals and derived hemodynamic metrics (Supplementary Tables S1-6). We focused on hemodynamic monitoring, especially cardiac output (Supplementary Table S1), but also gathered data on cardiac index (Supplementary Table S2), estimated patch data during exercise such as stroke volume, heart rate, and systemic vascular resistance (Supplementary Table S3) compared to RHC data during exercise (Supplementary Table S4), blood pressure (Supplementary Table S5), and other general vital signs such as respiratory rate, oxygen saturation, and temperature (Supplementary Table S6).

Cardiac output at rest ranged from 4.0 to 5.8 L/min (mean 5.0) by the patch and 4.3 to 7.1 L/min (mean 5.8) by RHC. During exercise, cardiac output increased independent of the measurement mode: at passive leg raise (patch mean 5.4; RHC mean 6.0) and at 20 W (patch mean 7.0; RHC mean 7.9). Heart rate increased from a mean of 68 bpm at rest to 72 bpm at leg raise and 87 bpm at 20 W, and therefore mainly seems to influence cardiac output in this group, while patch-derived stroke volume showed a smaller increase (rest mean 75 ml; 20 W mean 81 ml).

### Exercise measurements—statistical analysis

**Fig. 2:**
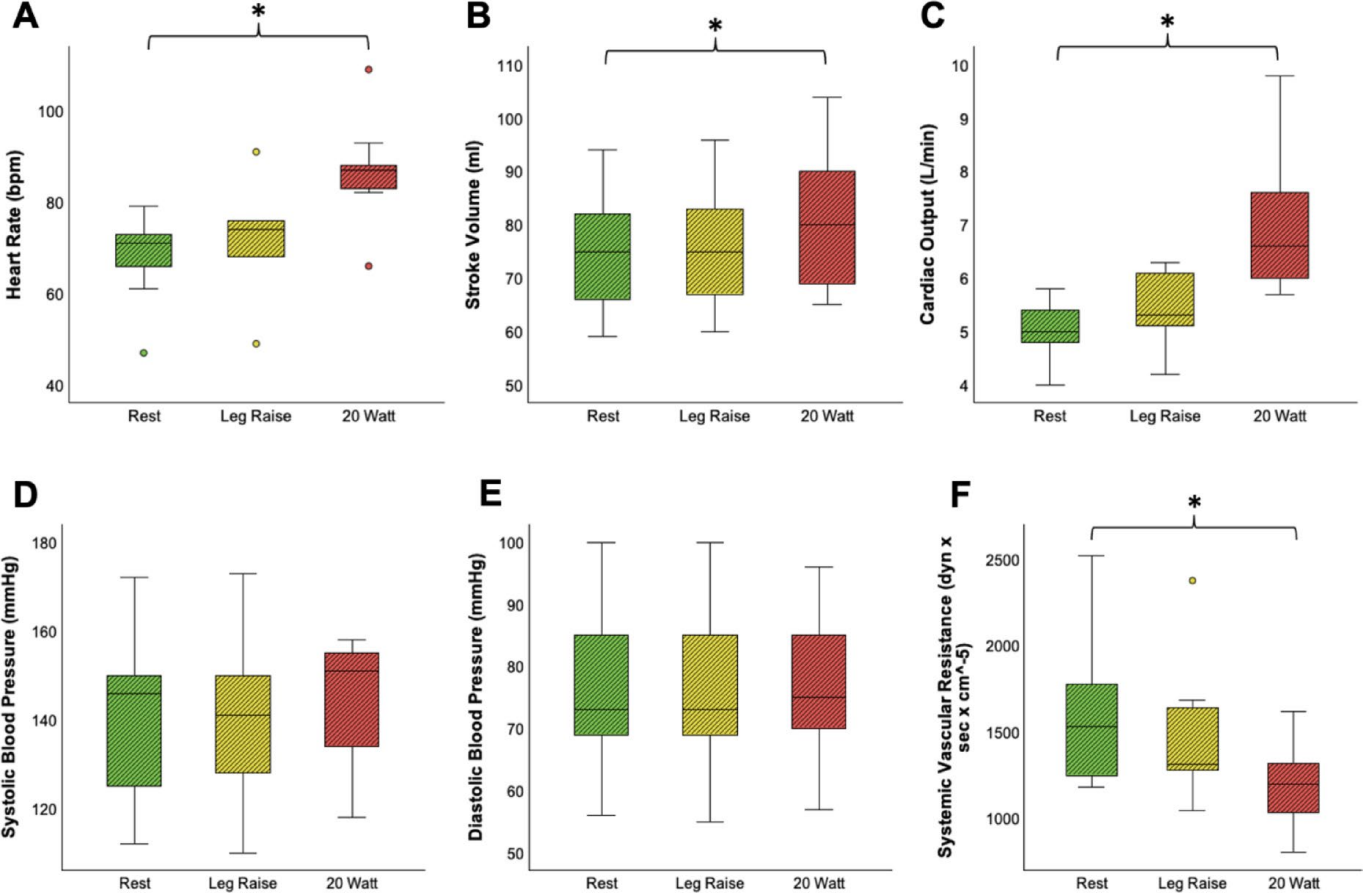
Hemodynamic monitoring using a chest patch showing significant increases in heart rate (**A**), stroke volume (**B**), and cardiac output (**C**) at rest and at 20 Watt exercise level. Measurements of systolic (**D**) and diastolic (**E**) blood pressure and systemic vascular resistance (**F**) at different exercise levels showed a significant decrease of systemic vascular resistance when comparing rest and 20 Watt exercise levels. All patch-derived values shown here (heart rate, stroke volume, cardiac output and systemic vascular resistance) are algorithm-based estimates, not direct measurements.

Heart rates increased during exercise (rest 67.7 ± 9.3 bpm vs 20 W 86.7 ± 11.2 bpm; *p* < 0.001), as assessed by the PPG patch. Patch-derived stroke volume also increased from 74.9 ml at rest to 81.3 ml at 20 W (*p* < 0.001) (Fig. 2A-C). Mean CO at rest was 5.0 ± 0.6 L/min (patch) and 5.8 ± 1.1 L/min (thermodilution); both increased at 20 W (patch 7.0 ± 1.4 L/min, *p* < 0.001; thermodilution 7.9 ± 2.1 L/min, *p* = 0.002). Patch-derived systemic vascular resistance decreased significantly (rest 1586 ± 418 vs. 20 W 1177 ± 271 dyn s cm^-5^; *p* < 0.001) (Fig. 2D-F).

**Fig. 3:**
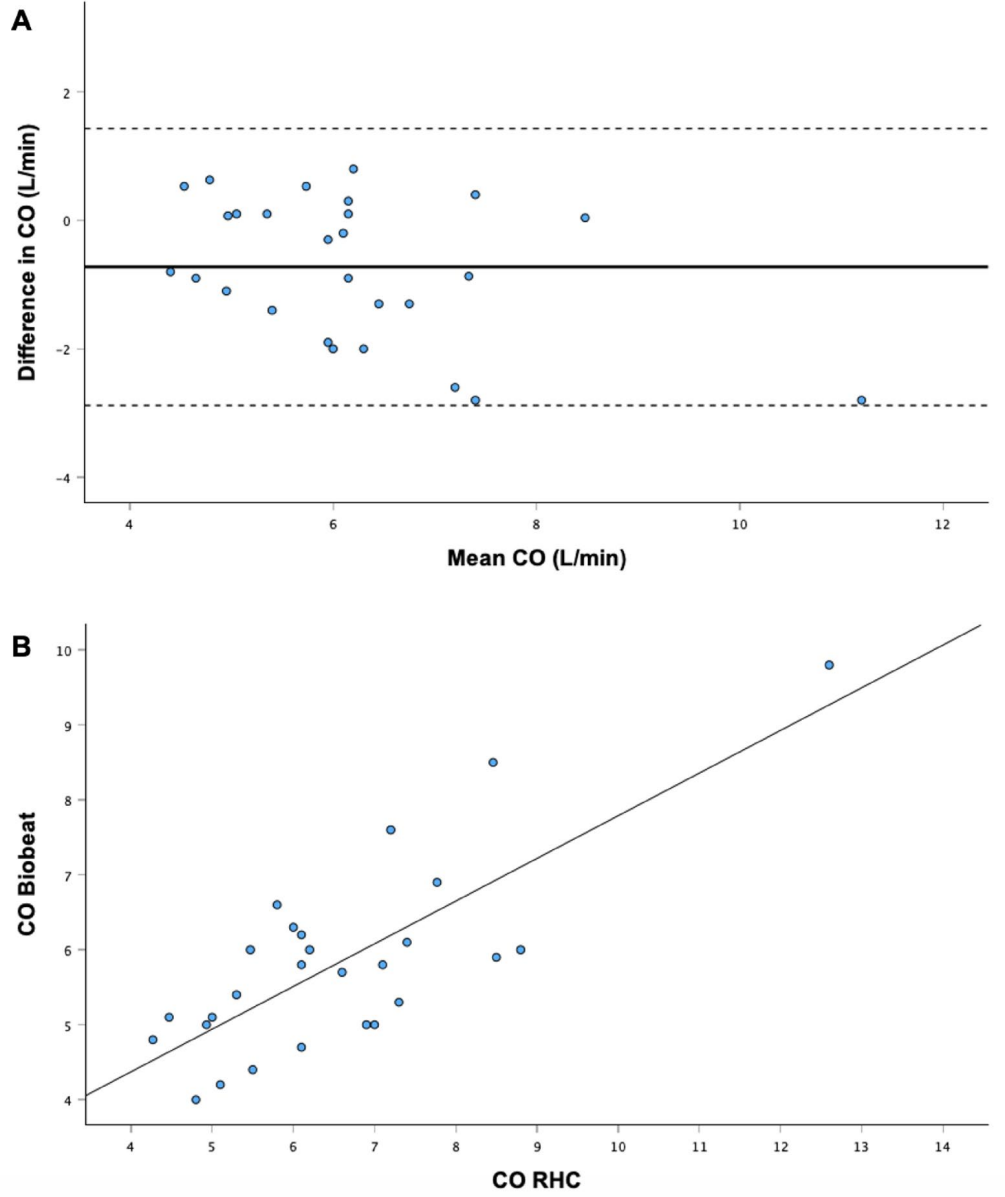
(**A**) Bland–Altman plot comparing chest patch data with right heart catheterization data. Bland–Altman analysis showed a bias of -0.72 L/min with –2.88 and 1.43 L/min 95% limit of agreement for the chest-patch-derived cardiac output (CO) estimates. The negative bias suggests a systematic underestimation of cardiac output by the patch relative to thermodilution. (**B**) Correlation analysis between CO measured during exercise with right heart catheterization and a chest patch sensor. The CO values obtained by thermodilution and chest patch were highly correlated (r = 0.774; *p* < 0.001).

The Bland–Altman analysis showed a bias of -0.72 L/min (95% CI − 1.16 to − 0.29) with 95% limits of agreement of − 2.88 to 1.43 L/min for the PPG-patch-derived CO measurements (Fig. 3A). Using the method for multiple observations per subject, the bias was unchanged (− 0.72 L/min) and the repeated-measures-corrected SD of the differences was 1.13 L/min, giving 95% limits of agreement of approximately − 2.95 to + 1.50 L/min. The overall percentage error was 33% (relative to the mean thermodilution cardiac output). The CO values obtained by thermodilution and the PPG patch were strongly correlated (r = 0.774; *p* < 0.001) (Fig. 3B).

Looking at the differences (patch minus thermodilution) on the means demonstrated a statistically significant negative slope (slope − 0.34; *p* = 0.021), indicating proportional bias with increasing underestimation at higher cardiac outputs. Stage-specific Bland–Altman analyses showed a bias of − 0.74 L/min (LoA − 2.52 to + 1.03; percentage error 31%) at rest, -0.52 L/min (LoA − 2.30 to + 1.26; 30%) at passive leg raise, and − 0.91 L/min (LoA − 3.82 to + 2.00; 37%) at 20 W; agreement was therefore best at rest and at passive leg raise and widened during exercise (Table 2**, **Fig. 4). In the change analysis, the patch tracked the thermodilution-derived changes in cardiac output well, with a concordance of r = 0.88 (*p* = 0.002) for the rest-to-20 W change (mean change + 1.98 L/min for the patch vs + 2.14 L/min for thermodilution; bias of the change -0.17 L/min, LoA -1.59 to + 1.26) and r = 0.81 (*p* = 0.008) for the leg-raise-to-20 W change.

**Table 2:** Stage-specific Bland–Altman results at rest, leg raise and 20 Watt. LoA = limits of agreement; PE = percentage error.

Stage-specific Bland–Altman results			
	Rest	Leg raise	20 Watt
Bias	0.74 L/min	− 0.52 L/min	− 0.91 L/min
LoA	− 2.52 to + 1.03 L/min	− 2.30 to + 1.26 L/min	− 3.82 to + 2.00 L/min
PE	30.7%	29.8%	36.8%

**Figure:**
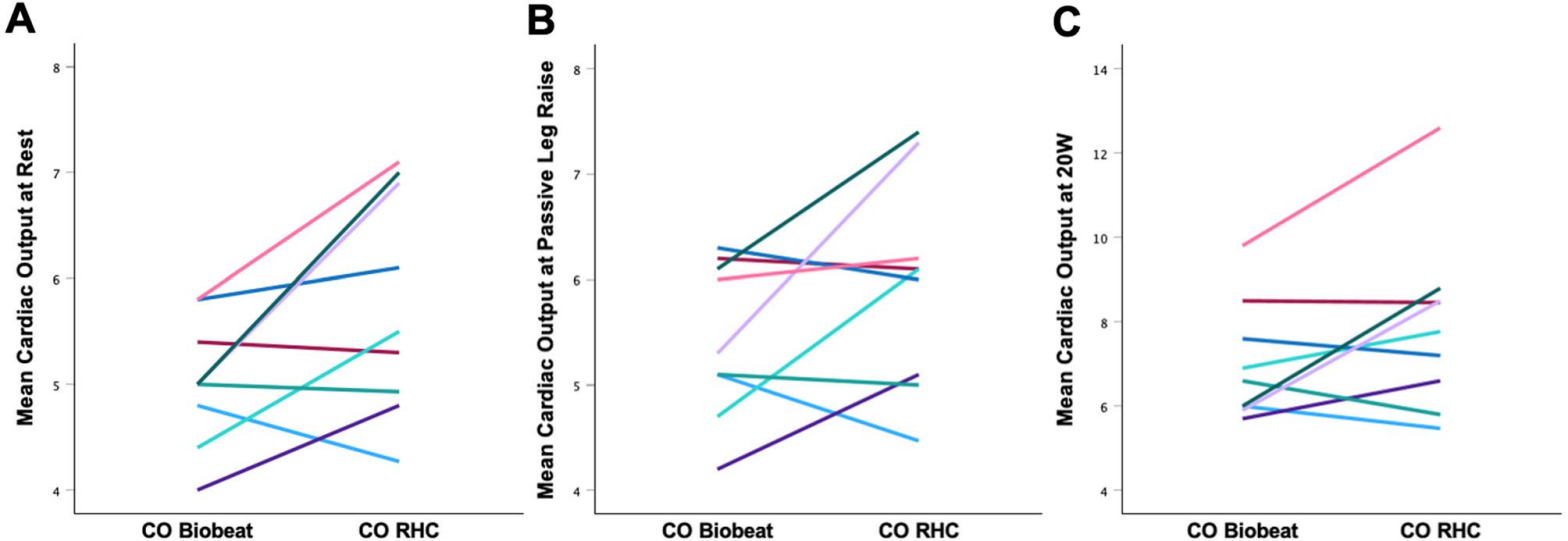
Paired patch and thermodilution cardiac-output values at rest (**A**), at passive leg raise (**B**) and at 20 W (**C**) shown in paired dot plot with individual lines. CO = cardiac output; RHC = right heart catheterization.

## Discussion

In this single-center, observational feasibility study, we compared the assessment of cardiac output during bicycle exercise measured by invasive RHC and a noninvasive chest patch sensor. Nine patients yielded 27 paired measurements. The CO values obtained using RHC and the PPG patch were strongly correlated (r = 0.774; *p* < 0.001) (Fig. 3B).

Remote monitoring has been established in various domains of heart failure management. Golbus et al. showed that smartwatch step counts correlated with health status^28^; Stehlik et al. showed that noninvasive remote monitoring can predict rehospitalization comparably to implanted devices^29^; and PPG-based wearables have shown promise for early detection of deterioration^19^ and for guiding diuretic therapy^20^.

Previous studies demonstrated PPG-based cardiac output monitoring in critically ill, typically ventilated and immobile, patients. Dvir et al. reported a low bias (0.2 L/min) and strong correlation (r = 0.906) against PiCCO^22^, and Nachman et al. reported an excellent correlation (r = 0.959) against pulmonary-artery-catheter CO in resting heart-failure patients^23^. Our lower correlation and wider limits of agreement are expected and are most plausibly explained by the setting: those studies were performed at rest in immobile patients. These conditions minimize motion artefact and respiratory and hemodynamic variability, whereas our measurements were obtained during physical exercise, which introduces motion artefact, increased respiratory variation and rapidly changing hemodynamics that degrade both the PPG signal and the precision of thermodilution.

Measuring hemodynamic parameters during exercise presents additional challenges. Nevertheless, our study demonstrates the feasibility of PPG-based chest monitoring during exercise, with heart rates rising significantly from rest to 20 W (67.7 ± 9.3 to 86.7 ± 11.2 bpm; *p* < 0.001). The 20 W stage was a fixed, protocol-defined sub-maximal workload analyzed for all patients rather than a symptom-limited maximal test, consistent with our diagnostic exercise RHC protocol for HFpEF. Cardiac output measurements during exercise using PPG and RHC correlated robustly (r = 0.774; *p* < 0.001). Our Bland–Altman analysis presented a higher bias (− 0.72 L/min) and wider limits of agreement (− 2.88 to 1.43 L/min) than prior resting studies^22,23^. The negative bias indicates a systematic underestimation of cardiac output by the patch relative to thermodilution, which the proportional-bias analysis shows is more pronounced at higher cardiac outputs. A change in cardiac output is generally considered clinically meaningful when it exceeds approximately 10–15% of baseline^30,31^, i.e. about 0.5–0.9 L/min for a physiological resting output of 5–6 L/min in a healthy adult; our bias fits in this order of magnitude and the limits of agreement are wide relative to it. Following Critchley and Critchley, a new technique is conventionally regarded as acceptable if the percentage error is below approximately 30%^24^; our overall value of 33% is marginally above this threshold. This benchmark, however, assumes a reference method with a precision of around 20%, whereas thermodilution during exercise is itself less precise than at rest, so a strict 30% criterion may be overly stringent in this setting. Taken together, the device tracks the direction and approximate magnitude of exercise-induced cardiac-output changes well (e.g. for the rest-to-20 W change (mean change + 1.98 L/min for the patch vs + 2.14 L/min for thermodilution; bias of the change -0.17 L/min, LoA -1.59 to + 1.26). But the absolute bias, limits of agreement and percentage error show wide differences between RHC and patch measurements. Although taking inaccuracies of the RHC measurements itself during exercise into account as well, patch data needs to be interpreted with caution, potentially underestimating the CO. Thermodilution was selected as the reference because it is the routinely used method during clinical exercise RHC at our center and provides discrete measurements that can be time-aligned with the exercise stages; direct Fick cardiac output was not measured. Thermodilution and direct Fick are not interchangeable, and published comparisons report clinically relevant discrepancies between these two invasive methods^32^, particularly during exercise, which demonstrates a potential frame of reference for interpreting the agreement we observed between the patch and thermodilution.

To our knowledge, this is the first study to investigate advanced hemodynamic parameters using a remote PPG-based wearable device during exercise. This approach offers cost efficiency, ease and speed of use, patient safety through non-invasiveness, and independence from operator variability, and opens potential for future artificial-intelligence-based analysis. We position the wearable as a complementary, low-risk tool for non-invasive flow monitoring rather than a replacement for the pressure-based invasive assessment required for HFpEF diagnosis.

### Limitations

Several limitations must be considered. This was a single-center study with a small cohort. With only nine patients, the limits of agreement are estimated imprecisely, and correlation coefficients are unstable and prone to inflation in small samples, so we rely primarily on agreement statistics rather than correlation for our conclusions. Exercise data were acquired during supine bicycle ergometry, which may limit generalizability to other forms of exercise. The cohort was also selected: eight of nine patients were overweight or obese (BMI ≥ 25 kg/m^2^) and had pulmonary hypertension, so the findings may not generalize to lean HFpEF phenotypes or to HFpEF patients without pulmonary hypertension; body habitus may itself influence the quality of the chest PPG signal. The patch does not provide intracardiac or pulmonary pressures, and pressure-based parameters central to HFpEF diagnosis (in particular pulmonary capillary wedge pressure) and tissue characterization by endomyocardial biopsy cannot be obtained or validated with this device. The stroke-volume values are patch-derived estimates that were not independently validated against an invasive reference in this study, and the device algorithm is proprietary and cannot be reconstructed by the reader. Moreover, because heart rate was not consistently documented in the RHC reports and thermodilution yields cardiac output without a directly measured stroke volume, a reference stroke volume could not be reconstructed for the RHC data, so the patch-derived stroke volume could not be compared component-by-component with the invasive reference. Currently, PPG patches have only been validated for inpatient use. Potential bias may have arisen from missing data due to impaired wireless transmission or temporal misalignment between RHC and patch measurements. Larger trials are needed to further validate the role of wearable technology in heart failure management and interventional cardiology.

Our findings suggest that wearable monitoring during exercise is feasible and tracks exercise-induced changes in cardiac output well, suggesting a potential avenue for safer and broader hemodynamic assessment in patients with heart failure, although confirmation in a larger validation cohort is required before clinical adoption. Future studies should focus on expanding sample sizes, refining device algorithms, and exploring outpatient and ambulatory settings.

## Supplementary Information

Below is the link to the electronic supplementary material.

Supplementary Material 1

## Data Availability

All data supporting the findings of this study are available within the paper and its Supplementary Information. The data used to calculate the results of this study are available upon request from the corresponding author.

## Abbreviations

BMI: Body mass index
CO: Cardiac output
ESC: European Society of Cardiology
HFpEF: Heart failure with preserved ejection fraction
LoA: Limits of agreement
PCWP: Pulmonary capillary wedge pressure
PE: Percentage error
PiCCO: Pulse contour cardiac output
PPG: Photoplethysmography
RHC: Right heart catheterization
