# Supplementary material for: Feasibility of a noninvasive photoplethysmography-based wearable chest patch for cardiac output assessment during exercise compared with thermodilution right heart catheterization
Source: Sci Rep. 2026 Jul 22;16:22946. doi: 10.1038/s41598-026-63092-z (PMC13392229; doi:10.1038/s41598-026-63092-z)
Supplement: Supplementary file 1 — Supplementary Material 1 [file 41598_2026_63092_MOESM1_ESM.docx]

**Feasibility of a Noninvasive Photoplethysmography-based Wearable Chest Patch**

**for Cardiac Output Assessment During Exercise**

**Compared with Thermodilution Right Heart Catheterization**

Bernd Stücker^1^, Alexander Hoss^1^, Jafer Haschemi^1^, Fabian Voß^1^, Amin Polzin^1,2^,

Malte Kelm^1,2^, Christian Jung^1,2^, Oliver Maier*^1^

Supplementary Material

**SUPPLEMENTARY FIGURES**

**
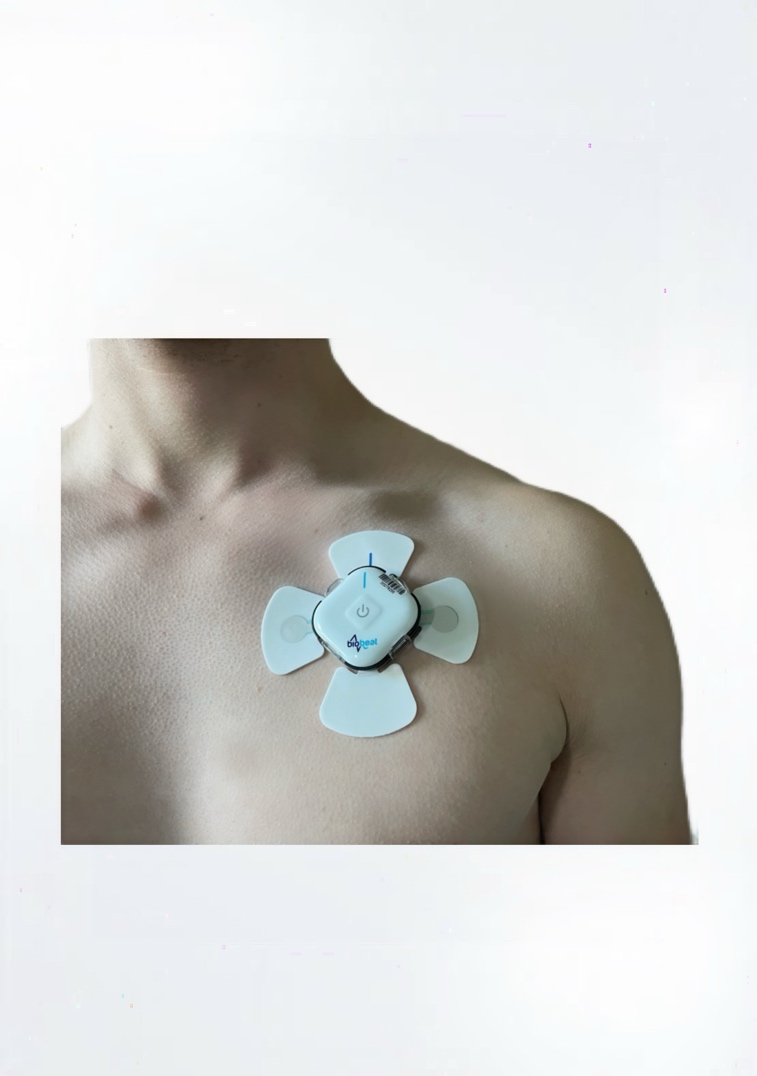

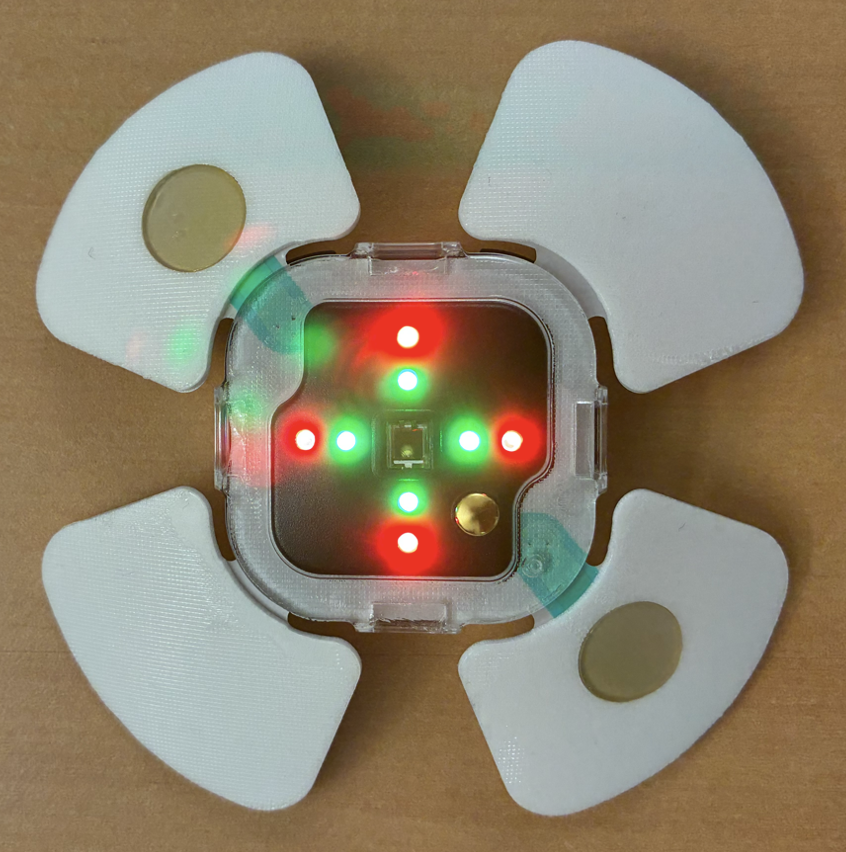
**

**Supplementary Figure S1.** Biobeat chest patch. The plethysmography-based monitor was placed on the left upper chest wall.


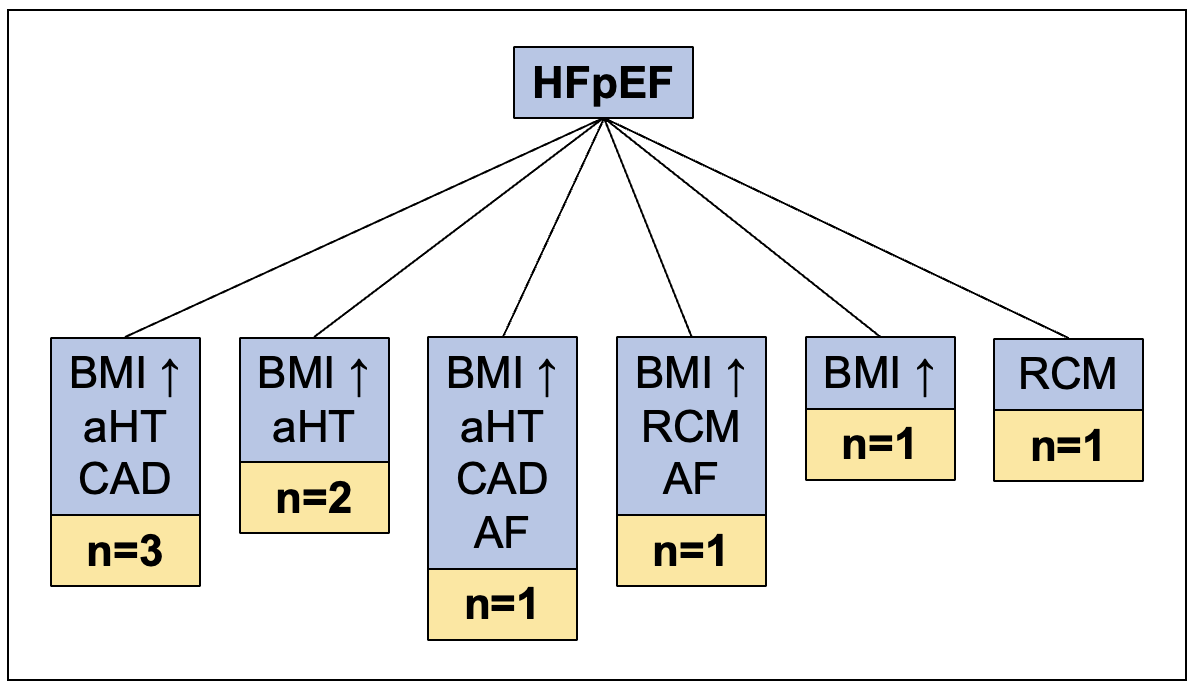


**Supplementary Figure S2**. Clinical entities of HFpEF. In our study group, obesity (89%) and arterial hypertension (67%) were the main drivers. AF = atrial fibrillation; aHT = arterial hypertension; BMI = body mass index; CAD = coronary artery disease; HFpEF = heart failure with preserved ejection fraction; RCM = restrictive cardiomyopathy.

# SUPPLEMENTARY TABLES

| **Cardiac Output (L/min)** | | | | | | |
| --- | --- | --- | --- | --- | --- | --- |
| **#** | **Rest** | | **Leg Raise** | | **20 Watt** | |
|  | **Patch** | **RHC** | **Patch** | **RHC** | **Patch** | **RHC** |
| 1 | 4.80 | 4.27 | 5.10 | 4.47 | 6.00 | 5.47 |
| 2 | 5.40 | 5.30 | 6.20 | 6.10 | 8.50 | 8.46 |
| 3 | 4.40 | 5.50 | 4.70 | 6.10 | 6.90 | 7.77 |
| 4 | 4.00 | 4.80 | 4.20 | 5.10 | 5.70 | 6.60 |
| 5 | 5.00 | 4.93 | 5.10 | 5.00 | 6.60 | 5.80 |
| 6 | 5.80 | 6.10 | 6.30 | 6.00 | 7.60 | 7.20 |
| 7 | 5.00 | 6.90 | 5.30 | 7.30 | 5.90 | 8.50 |
| 8 | 5.00 | 7.00 | 6.10 | 7.40 | 6.00 | 8.80 |
| 9 | 5.80 | 7.10 | 6.00 | 6.20 | 9.80 | 12.60 |

**Supplementary Table S1.** Exercise estimations (patch) and measurements (RHC) of cardiac output (L/min). RHC = right heart catheterization.

| **Cardiac Index (L/min/m^2^)** | | | | | | |
| --- | --- | --- | --- | --- | --- | --- |
| **#** | **Rest** | | **Leg Raise** | | **20 Watt** | |
|  | **Patch** | **RHC** | **Patch** | **RHC** | **Patch** | **RHC** |
| 1 | 2.80 | 2.50 | 3.00 | 2.60 | 3.50 | 3.22 |
| 2 | 2.70 | 2.71 | 3.10 | 3.12 | 4.30 | 4.33 |
| 3 | 1.90 | 2.44 | 2.00 | 2.71 | 3.00 | 3.45 |
| 4 | 2.20 | 2.70 | 2.30 | 2.90 | 3.10 | 3.70 |
| 5 | 3.40 | 3.38 | 3.40 | 3.42 | 4.40 | 3.97 |
| 6 | 2.90 | 3.10 | 3.10 | 3.10 | 3.80 | 3.60 |
| 7 | 2.50 | 3.50 | 2.60 | 3.70 | 3.00 | 4.30 |
| 8 | 2.00 | 3.27 | 2.80 | 3.45 | 2.90 | 4.10 |
| 9 | 2.60 | 3.30 | 2.70 | 2.80 | 4.40 | 5.70 |

**Supplementary Table S2.** Exercise estimations (patch) and measurements (RHC) of the cardiac index (L/min/m^2^). RHC = right heart catheterization.

| **Stroke Volume (ml), Heart Rate (bpm)**  **& Systemic Vascular Resistance (dyn x sec x cm^-5^)** | | | | | | | | | |
| --- | --- | --- | --- | --- | --- | --- | --- | --- | --- |
| **#** | **Rest** | | | **Leg Raise** | | | **20 Watt** | | |
|  | **SV** | **HR** | **SVR** | **SV** | **HR** | **SVR** | **SV** | **HR** | **SVR** |
| 1 | 66 | 73 | 1244 | 67 | 76 | 1162 | 69 | 85 | 1061 |
| 2 | 87 | 61 | 1180 | 90 | 68 | 1045 | 97 | 88 | 806 |
| 3 | 94 | 47 | 1795 | 96 | 49 | 1685 | 104 | 66 | 1197 |
| 4 | 59 | 66 | 2520 | 60 | 70 | 2378 | 65 | 87 | 1622 |
| 5 | 75 | 67 | 1532 | 75 | 68 | 1519 | 80 | 83 | 1196 |
| 6 | 82 | 71 | 1378 | 83 | 76 | 1279 | 87 | 87 | 1032 |
| 7 | 69 | 72 | 1778 | 71 | 75 | 1642 | 73 | 82 | 1492 |
| 8 | 63 | 79 | 1596 | 66 | 91 | 1311 | 67 | 93 | 1321 |
| 9 | 79 | 73 | 1248 | 80 | 74 | 1313 | 90 | 109 | 864 |

**Supplementary Table S3.** Estimated patch data during exercise. SV = stroke volume (ml); HR = heart rate (beats per minute); SVR = systemic vascular resistance (dyn x sec x cm^-5^).

| **Right Atrial Pressure, Mean Arterial Pressure, Cardiac Output**  **& Calculated Systemic Vascular Resistance during RHC** | | | | | | | | | | | | |
| --- | --- | --- | --- | --- | --- | --- | --- | --- | --- | --- | --- | --- |
| **#** | **Rest** | | | | **Leg Raise** | | | | **20 Watt** | | | |
|  | **RAP** | **MAP** | **CO** | **SVR** | **RAP** | **MAP** | **CO** | **SVR** | **RAP** | **MAP** | **CO** | **SVR** |
| 1 | 12 | 77 | 4.27 | 1217 | 14 | 91 | 4.47 | 1374 | 17 | 93 | 5.47 | 1111 |
| 2 | 10 | 77 | 5.30 | 1019 | 11 | 91 | 6.10 | 1049 | 12 | 93 | 8.46 | 766 |
| 3 | 3 | 100 | 5.50 | 1411 | 4 | 101 | 6.10 | 1279 | 5 | 105 | 7.77 | 1030 |
| 4 | 8 | 101 | 4.80 | 1550 | 12 | 102 | 5.10 | 1412 | 12 | 92 | 6.60 | 970 |
| 5 | 7 | 98 | 4.93 | 1479 | 6 | 104 | 5.00 | 1568 | 10 | 94 | 5.80 | 1159 |
| 6 | 20 | 104 | 6.10 | 1102 | 25 | 104 | 6.00 | 1053 | 30 | 122 | 7.20 | 1022 |
| 7 | 13 | 103 | 6.90 | 1043 | 18 | 106 | 7.30 | 964 | 20 | 115 | 8.50 | 894 |
| 8 | 8 | 89 | 7.00 | 926 | 9 | 96 | 7.40 | 941 | 12 | 97 | 8.80 | 773 |
| 9 | 17 | 94 | 7.10 | 868 | 17 | 102 | 6.20 | 1097 | 20 | 106 | 12.60 | 546 |

**Supplementary Table S4.** RHC data during exercise. CO = cardiac output (L/min); MAP = calculated mean arterial pressure (mmHg); RAP = right atrial pressure (mmHg); RHC = right heart catheterization; SVR = calculated systemic vascular resistance (dyn*s*cm^-5^).

| **Systolic, Diastolic & Mean Arterial Pressure from Patch (mmHg)** | | | | | | | | | |
| --- | --- | --- | --- | --- | --- | --- | --- | --- | --- |
| **#** | **Rest** | | | **Leg Raise** | | | **20 Watt** | | |
|  | **SBP** | **DBP** | **MAP** | **SBP** | **DBP** | **MAP** | **SBP** | **DBP** | **MAP** |
| 1 | 112 | 56 | 74 | 110 | 55 | 74 | 118 | 57 | 78 |
| 2 | 119 | 58 | 78 | 122 | 59 | 80 | 134 | 62 | 86 |
| 3 | 150 | 73 | 99 | 150 | 73 | 99 | 158 | 75 | 103 |
| 4 | 172 | 100 | 124 | 173 | 100 | 124 | 155 | 96 | 115 |
| 5 | 150 | 69 | 96 | 149 | 69 | 95 | 155 | 70 | 99 |
| 6 | 155 | 73 | 100 | 155 | 73 | 100 | 151 | 72 | 98 |
| 7 | 146 | 92 | 110 | 141 | 91 | 108 | 147 | 92 | 110 |
| 8 | 128 | 85 | 100 | 128 | 85 | 99 | 127 | 85 | 99 |
| 9 | 125 | 73 | 90 | 138 | 76 | 97 | 156 | 80 | 105 |
| **Systolic, Diastolic & Mean Arterial Pressure during RHC (mmHg)** | | | | | | | | | |
| **#** | **Rest** | | | **Leg Raise** | | | **20 Watt** | | |
|  | **SBP** | **DBP** | **MAP** | **SBP** | **DBP** | **MAP** | **SBP** | **DBP** | **MAP** |
| 1 | 118 | 57 | 77 | 133 | 70 | 91 | 114 | 82 | 93 |
| 2 | 118 | 57 | 77 | 133 | 70 | 91 | 114 | 82 | 93 |
| 3 | 139 | 81 | 100 | 154 | 74 | 101 | 156 | 80 | 105 |
| 4 | 164 | 70 | 101 | 161 | 72 | 102 | 155 | 60 | 92 |
| 5 | 153 | 70 | 98 | 148 | 82 | 104 | 122 | 80 | 94 |
| 6 | 162 | 75 | 104 | 154 | 79 | 104 | 153 | 106 | 122 |
| 7 | 160 | 75 | 103 | 169 | 75 | 106 | 171 | 87 | 115 |
| 8 | 130 | 69 | 89 | 136 | 76 | 96 | 155 | 68 | 97 |
| 9 | 133 | 75 | 94 | 141 | 82 | 102 | 141 | 88 | 106 |

**Supplementary Table S5.** Blood pressure measurements during exercise. Data estimated by the patch shown above; data taken during RHC shown below (arterial pressure during RHC was measured manually by cuff). DBP = diastolic blood pressure (mmHg); MAP = (calculated) mean arterial pressure (mmHg); RHC = right heart catheterization; SBP = systolic blood pressure (mmHg).

| **Respiratory Rate (breaths per minute), Oxygen Saturation (%)**  **& Temperature (°C)** | | | | | | | | | |
| --- | --- | --- | --- | --- | --- | --- | --- | --- | --- |
| **#** | **Rest** | | | **Leg Raise** | | | **20 Watt** | | |
|  | **RR** | **SO2** | **Temp** | **RR** | **SO2** | **Temp** | **RR** | **SO2** | **Temp** |
| 1 | 16 | 98 | 36.4 | 16 | 99 | 36.5 | 22 | 99 | 36.5 |
| 2 | 16 | 99 | 36.5 | 15 | 99 | 36.5 | 18 | 99 | 36.5 |
| 3 | 13 | 98 | 36.4 | 14 | 97 | 36.4 | 20 | 99 | 36.4 |
| 4 | 16 | 97 | 36.5 | 15 | 98 | 36.5 | 18 | 99 | 36.4 |
| 5 | 17 | 99 | 36.4 | 17 | 98 | 36.4 | 17 | -- | 36.4 |
| 6 | 18 | 95 | 36.8 | 18 | 99 | 36.8 | 19 | 96 | 36.7 |
| 7 | 18 | -- | 36.5 | 19 | 99 | 36.5 | 17 | -- | 36.5 |
| 8 | 11 | 94 | 37.2 | 13 | 96 | 37.2 | 12 | 91 | 37.2 |
| 9 | 14 | 99 | 37.4 | 20 | 99 | 37.3 | 17 | 93 | 36.9 |

**Supplementary Table S6.** Wearable patch data during exercise. RR = respiratory rate (breaths per minute); SO2 = oxygen saturation (%); Temp = body temperature (°C).
